# Technological research of process for producing titanium rich slag and complex titanium-containing ferroalloy

**DOI:** 10.1016/j.heliyon.2023.e18989

**Published:** 2023-08-06

**Authors:** Nina Vorobkalo, Alibek Baisanov, Yerbolat Makhambetov, Yesmurat Mynzhasar, Nurzhan Nurgali

**Affiliations:** a Zh. Abishev Chemical-Metallurgical Institute, Laboratory of Pyrometallurgical Processes, Karaganda, Kazakhstan; b NPJSC “Abylkas Saginov Karaganda Technical University”, Kazakhstan; c Zh. Abishev Chemical-Metallurgical Institute, Laboratory of Ferroalloys and Recovery Processes, Karaganda, Kazakhstan; d ERG Research and Engineering Center, LLP, Astana, Kazakhstan

**Keywords:** Titanium, Titanium rich slag, Titanium-containing ferroalloy, Ore-thermal furnace, Smelting, Carbothermal process, Metals and alloys

## Abstract

This paper demonstrates the results on the experimental smelting of the titanium rich slag and complex titanium-containing ferroalloy under the large-scale laboratory conditions that simulate industrial one. The technological researches of the process were performed on an ore-thermal furnace with 200 kVA transformer power. The titanium rich slag was produced from the low-grade ilmenite concentrate, i.e. the low TiO_2_ content and the high content of impurities. During the production of the high-grade titanium slag (TiO_2_ content: 75–80%), the impurity elements are transferred into the associated alloyed metal (cast iron). Thus, it can be used to smelt the steel. As a result, samples of titanium slag have been produced with the content of the main components, %: TiO_2_ – 80.2; Al_2_O_3_ – 4.5; SiO_2_ – 1.97; Cr_2_O_3_ – 1.3 and Fe_2_O_3_ – 9.87. Then, in metallurgical practice a complex titanium-containing ferroalloy was first smelted from the previously produced titanium rich slag using a carbothermic approach. The high-ash coal was applied as a carbon-bearing reducing agent. The ash was more 45%. As a result of tests, a pilot batch of the alloy was produced with the following chemical composition, %: Ti - 20–25; Si - 35–45; Al - 10–15; C - 0.2–0.5; P - no more than 0.08; and ferrum. The main component content in the produced alloy suggests that it can serve as an alternative to a mechanical mixture (FeSi45, aluminum shavings, low-percentage ferrotitanium) for steel alloying and deoxidation purposes.

## 1 Introduction

A potential for widespread use of titanium and titanium-containing products is directly related to the fact that titanium-containing alloys have a sufficiently high tensile strength and fracture toughness. In addition to lightness, titanium alloys can tolerate a wide range of temperatures. They have the especial corrosion resistance equal to nickel and copper alloys, etc. This feature makes titanium one of the key elements in the architecture, the chemical, automotive and aerospace industries [[1], [2], [3], [4]].

Titanium rich slag is the main raw material to produce titanium sponge and titanium dioxide pigment (titanium white) [5]. Sponge titanium is used to produce titanium alloys with the high physical and chemical properties for the mechanical engineering, aircraft construction, rocket and nuclear power engineering, and for other strategically important industries [[6], [7], [8], [9], [10]].

Currently, ferrotitanium is a key material used for alloying, deoxidizing and degassing the steel with titanium. Titanium in steel is active carbide former. It is added in small amounts (0.02–0.5%) to bind carbon in manganese-, chromium-, chromium-molybdenum-, and chromium-nickel stainless steels. Thus, it eliminates intergranular corrosion, carbides and crushing of the structure of steel castings [11].

Titanium binds sulfur into strong sulfides, i.e. it prevents the steel structures and products from defect of hot-brlttleness [12]. Based on the above, it found its wide application in the ferrous metallurgy. This largely predetermines its overall demand and volume of its production [13].

The main raw material to produce titanium-containing products is titanium-containing ores and concentrates. Now, the world raw material base of ferrum -titanium ores, including ilmenite ores, is represented by a number of powerful deposits. They can supply the raw materials to the metallurgical enterprises for hundreds of years. Based on the current estimates, the global reserves of titanium oxide amount to 650 billion metric tons, including minerals of rutile (TiO_2_) and ilmenite (FeTiO_3_) are widely used to extract various titanium-containing products using the electric furnace smelting and the hydrometallurgical processes [14,15]. Ilmenite is an important mineral [16,17] that contains 40%–65% of titanium dioxide. Other elements are ferrous oxide, and sometimes they have small amounts of vanadium, chromium, magnesium and/or manganese. Currently, ilmenite accounts for 92% of the global production of the titanium mineral. Rutile (TiO_2_) has a titanium dioxide content of 93–96%. However, it is difficult to find in the natural ilmenite deposits [18].

In the world, about half of the exploited titanium placers are ilmenite placers. The primary ilmenite deposits are also exploited. The largest reserves of ilmenite are in China (the magmatic deposits), Australia, India, South Africa, Norway, USA, Canada, Mozambique, Ukraine, Brazil and Vietnam.

Currently, the raw material base of the ferrum - titanium ores, including ilmenite in the territory of the Republic of Kazakhstan is also represented by the large promising raw material deposits: Shokashsky deposit (Aktobe region), Satpaevsky deposit (East Kazakhstan region) and Obukhovsky deposit (Taiynshinsky district, North Kazakhstan region).

Titanium slag is produced traditionally from ilmenite concentrates in an ore-thermal electric furnace using the carbothermal reduction. Most of the ferrous oxides are converted into an associated alloyed metal. Titanium oxides and some impurity elements are converted into the slag [[19], [20], [21]].

Ferrotitanium with 20–40% of Ti is smelted mainly by an aluminothermic method of reducing the main alloying components: titanium and ferrum (oxides of concentrate) and titanomagnetite ores (ilmenite and rutile concentrate). The high-percentage grades are traditionally produced by alloying of titanium and ferrum scrap or steel cuttings in an induction furnace [22].

It is stated that today some approaches are known to produce ferrotitanium (30–55% of Ti) using titanium slag by the aluminothermic method [14,23]. One of the disadvantages to use aluminum as a reducing agent is its high price.

It is also noteworthy that aluminum (especially secondary aluminum) is a source for impurities of nonferrous metals, primarily lead. In ferrotitanium-alloyed steel, lead can degrade toughness of steel.

It is noted that the processing of titanium-containing concentrates and ores is difficult i.e. they are refractory and difficult to recover. In order to process such materials, it is necessary to create conditions for the primary solid-phase reduction of ferrum, i.e., the rate of reduction of ferrous oxides must run ahead the rate of formation of the slag phase and smelting of the charge. Complexity of this process imposes some specific requirements for the initial ilmenite concentrates. Concentrates must contain no more than 5% of impurity components difficult to recover, i.e., Al_2_О_3_, SiО_2_, CaO, MgO, MnO and Cr_2_O_3_. For instance, for concentrates, the current specifications limit Al_2_О_3_ content to 2.9%, SiO_2_ to 1.8%, and TiO_2_ to not less than 63.2% [24]. In concentrates, the ratio of titanium dioxide to ferrous oxides should be at 1:1.2. Otherwise, smelting and production of the high-grade titanium slag are made difficult.

The above mentioned deposits of ilmenite in the Republic of Kazakhstan do not meet the requirements for content of titanium dioxide and impurities. For this reason, Ust-Kamenogorsk Titanium–Magnesium Plant (Kazakhstan) uses a mixture of ilmenite concentrates of Volnogorsky deposit (66% of TiO_2_) imported from Ukraine, Shokashsky deposit (56.6% of TiO_2_) and Satpaevsky deposit (50.5 %-TiO_2_) to produce titanium rich slag.

However, in general, Ust-Kamenogorsk Titanium–Magnesium Plant has to use the finished imported titanium rich slag from Canada and Ukraine. Thus, it significantly increases the cost of the finished products (titanium sponge, titanium white). The content of impurity elements exceeds the allowable norm twice in the ilmenite concentrate of the Obukhovsky and Shokashsky deposits (in total is 10.6%). Moreover, the content of TiO_2_ is 52%, i.e. it is less by ∼8–10% of the allowable norm.

As part of our research it is planned to develop a technology to smelt TiO_2_-rich (more than 75%) titanium slag from substandard ilmenite concentrates by a carbothermic approach. The use of ilmenite concentrates in this technology does not imply the additional expensive stages of their enrichment. The developed technology proposes to transfer the impurity elements into the associated alloyed metal in the process of slag smelting by adding of the fluxing (diluting) additives.

The next objective of this research is to produce a complex titanium-containing ferroalloy, an analogue of low-percentage ferrotitanium, using titanium rich slag, where the substandard waste high ash coal will be applied as a reducing agent. Ash content is more than 45%.

## 2 Materials and methods

Tests to smelt titanium rich slag and, subsequently, a new complex titanium-containing ferroalloy (aluminum-silicon-titanium) from it were performed with using ilmenite concentrate from the Obukhovsky deposit (Kokshetau region, Republic of Kazakhstan).

Based on results of analysis on vacuum wave dispersive X-ray fluorescence spectrometer MAKS-GVM, ilmenite concentrate consists of 87.18% of titanium and ferrous oxides. It contains oxides of chromium, aluminum, manganese and silicon as impurities. The results of the spectral analysis are presented in Table 1.

**Table 1:** The chemical composition of ilmenite concentrate, mass %.

Material name	Component content, %							
	TiO_2_	SiO_2_	Fe_gener_	Al_2_O_3_	P	Cr_2_O_3_	MnO	MgO
Ilmenite concentrate	52.00–55.00	1.50–2.10	24.0–25.0	6.20–6.80	0.008	7.30–7.90	0.76	1.23

The chemical analysis demonstrates the high chromium content. It is known that high chromium content in ilmenite concentrate makes difficult to process into titanium-containing slag, titanium alloys, titanium tetrachloride, titanium sponge, etc. Some methods are known to reduce the chromium content in the concentrate. These methods include oxidizing roasting and magnetic separation [25,26]. However, the oxidizing roasting is very economically and operationally expensive. Thus, the developed technology removes chromium in the finished ilmenite concentrate during the carbothermic smelting for titanium rich slag. As a result, the unwanted impurities are converted into an associated alloyed metal to produce a high-grade titanium oxide product.

The charge mixture to smelt titanium rich slag consisted of the powdered ilmenite concentrate and various carbonaceous reducing agents (75% with the main charge and 25% for slag finishing).

Selection of the reducing agent plays a crucial role in carbothermic processes. For this purpose, various carbonaceous reducing agents were used to optimize the smelting of titanium rich slag.

For smelting of titanium rich slag, the low-ash reducing agents were used, i.e., some oxides (SiO_2_, Al_2_O_3_, CaО and MgO) convert into the slag and impoverish target product - slag. The low electrical conductivity and ash content of the reducing agent ensures a high electrical resistance of the charge, a deep fit of electrodes, a stable electrical mode and a uniform run of furnace. The special coke was applied as a reducing agent, which produced by the scientists of the Zh. Abishev Chemical-Metallurgical Institute using the developed technology [27]. The special coke is a solid carbonaceous reducing agent for the electrometallurgical production with the high reactivity, the specific electrical resistivity and with a low content of harmful impurities (sulfur and phosphorus) [28]. The technical and chemical compositions of the reducing agents are presented in Table 2, Table 3, respectively.

**Table 2:** The technical compositions of reducing agents, mass %.

Material name	C_solid_	A	V	W
Special coke	78.69	8.56	6.82	5.59
Low ash coal	45.84	2.47	45.13	6.56

**Table 3:** The chemical compositions of reducing agents, mass %.

Material name	SiO_2_	Al_2_O_3_	CaO	MgO	Р	S
Special coke	27.99	19.05	17.80	2.85	0.06	3.16
Low ash coal	34.41	24.71	11.86	0.46	0.03	2.04

The technical analysis of coals is represented by indicators describing the quality of coals, namely the content of solid carbon (C_solid_), ash content (A), volatile matter content (V) and moisture content (W). Volatile substances are a mixture of gas and vaporous substances released from coal as a result of the decomposition of organic matter during heating. Ash is represented by a mixture of mineral substances remaining after the combustion of all combustible parts.

The reducing agents were in amounts to completely recover the free and included ferrous oxides in the ilmenite concentrate, i.e., selective reduction.

The finished titanium rich slag will be applied to smelt the titanium-containing ferroalloy, analogue with low-percentage ferrotitanium grades.

In order to select the optimal charge mixture composition for the developed technology of titanium-containing ferroalloy production by the carbothermic method, the theoretical and experimental tests were performed [29,30].

Based on these tests, the most optimum alternatives of charge mixtures to produce one ton of titanium-containing ferroalloy have been calculated according to titanium content relevant to FeT30Al6 grade (ISO 5454–80). The charge was calculated for the complete reduction of all oxides.

Consumption of a reducing agent was calculated with an excess of solid carbon by 10% of stoichiometry, i.e., oxidation (burn-off) of a reducing agent was estimated with free oxygen of air [31].

In order to obtain a high-quality alloy and maintain a stable smelting mode in the ore-thermal furnace, the most optimal alternative of charge mixture is the chemical composition presented in Table 4 [32].

**Table 4:** The chemical composition of materials of the charge mixture.

Material		Share of materials in the charge mixture		The chemical composition of materials, %						
				TiO_2_	Al_2_O_3_	SiO_2_	Fe_gener_	Cr_2_O_3_	MnO	P
		%	tons							
1	Coal	67.35	2.4	1.00	26.85	60.32	1.55	0.20		0.15
	Rich titanium slag	26.12	0.9	80.10	4.50	1.97	6.90	1.30	3.59	–
	Quartzite	6.50	0.2	–	–	98.00	–	–	–	0.11

Reducing the share of quartzite (SiO_2_) in the total charge mixture (less than 6.5%) can form titanium carbide. Thus, it greatly disrupts the smelting process in the ore-thermal furnace. Therefore, the share of quartzite in the ore mixture should be not less 20%.

The technological researches of production process of the titanium rich slag and titanium-containing ferroalloy were performed on a 200 kVA large-sized laboratory arc single-phase electric furnace with graphite conductive hearth.

**Fig. 1:**
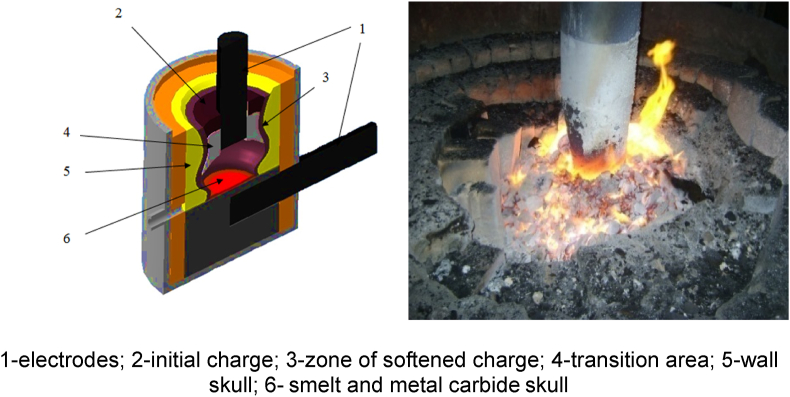
Structure of the ore-thermal furnace bath with 200 kVA transformer.

These tests simulate the industrial tests under the real production conditions. The graphical structure of the furnace bath is illustrated in Fig. 1.

The furnace casing in general form is a cylinder welded from 10 mm thick sheet steel. The electric furnace has the following geometric parameters (mm).

- casing diameter - 1400;
- bath diameter - 500;
- bath depth - 450;
- electrode diameter - 150;
- lining thickness - 400;
- distance from the electrode to the lining - 175;
- deepening of the electrode into the charge - 350.

Two 100 kVA furnace transformers connected in parallel provide a power of 200 kVA at a maximum voltage of 49 V and a current load of up to 4000 A. The electric furnace is equipped with four stages of secondary voltage regulation from 18.5 to 49.5 V. The temperature of the arc discharge is 2500–4500 °C. The furnace has two electrodes with a diameter of 150 mm. The lower electrode is rigidly coked in a hearth made of a conductive packing (carbon hearth mass). The upper electrode is fixed in a copper contact cheek placed on a current-insulated suspension. The movement of the electrode in the vertical plane is made manually through the gearbox. The control of the electrical mode is made through an ammeter connected to the high side of the furnace transformer. The bath of the furnace is stationary. It has one tap-hole equipped with a graphite tap-hole block. The furnace has a closed vault and is equipped with an exhaust hood located at a height of 700 mm from the upper cut of the furnace body. The process gas removal system is equipped with a cyclone and a wet scrubber to capture dust.

## 3 Theoretical description

### 3.1 Theoretical mechanism of rich titanium slag obtaining

The electric smelting process for obtaining rich titanium slag involves the use of ilmenite concentrate, It consists of metal oxides, and solid carbon as a reducing agent. The consumption of carbon was calculated specifically for the reduction of iron oxide, chromium, and other impurities, And the aim was to convert them into an associated alloyed metal and maximizing the conversion of TiO_2_ into slag. The nature of the processes involved in the formation of titanium slag and cast iron (the associated alloyed metal) during the electric melting depends on the interaction between the oxide components and carbon in solid or gaseous form, and their interaction with each other. Thus, probability of these reactions can be determined by the changes in their free Gibbs energy at different temperatures.

**Fig. 2:**
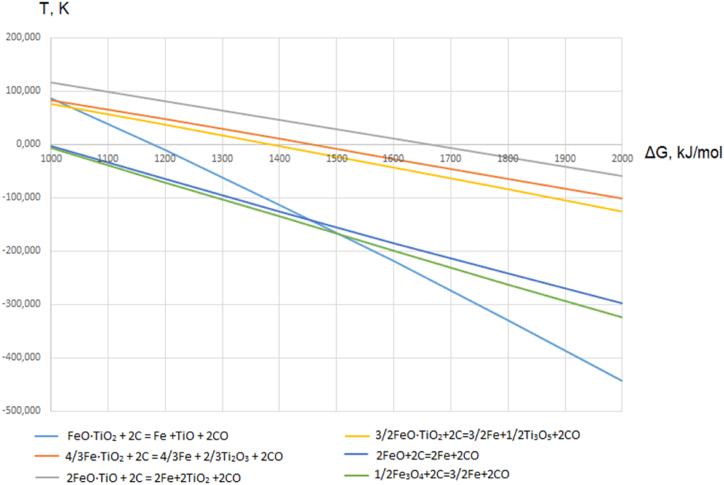
The dependence of the Gibbs free energy of ilmenite reduction reactions on temperature.

The dependence of the Gibbs free energy of ilmenite reduction reactions on temperature was plotted using the HSC Chemistry 12.0 software package. Based on this dependence (Fig. 2), it can be observed that hematite (Fe₂O₃) and magnetite (FeO·Fe_2_O_3_) are more readily reduced compared to ilmenite (FeO·TiO_2_). The reduction of ilmenite with formation of free iron is only possible at temperatures close to 1000 °C. In addition, iron oxides, titanium dioxide is also reduced, leading to formation of lower oxides. Reduction of pure TiO_2_ with carbon in the presence of CO_2_ demostrates the appearance of Ti_2_O_3_ at 870 °C. The reduction of TiO2 begins at 1100 °C with participation of iron oxides. Formation of lower titanium oxides can be represented by the following scheme of the lower titanium oxides:

TiO_2_ → TiO_х_ → (Ti_n-1_ O_2n-1_ … Ti_4_O_7_) → Ti_3_O_5_→Ti_2_O_3_→TiO→Ti

Ti_3_O_5_, Ti_2_O_3_, TiO are considered the most stable, their formation proceeds sequentially.

### 3.2 Theoretical mechanism of complex titanium-containing ferroalloy obtaining

**Fig. 3:**
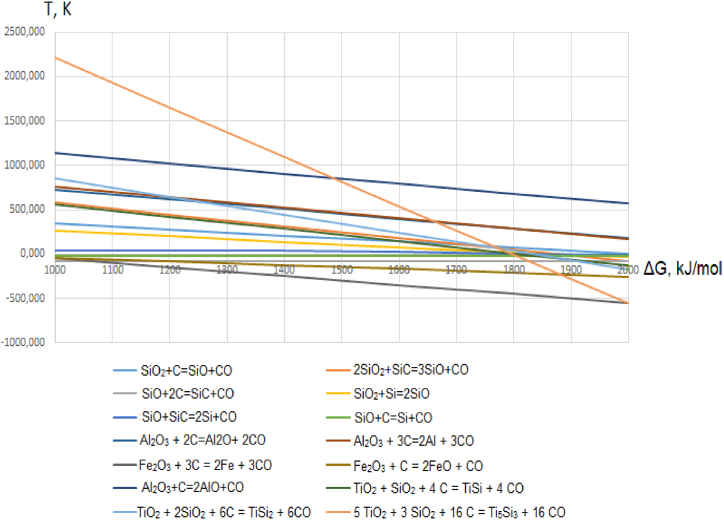
The dependence of the Gibbs free energy of reduction reactions during the smelting of a new complex titanium-containing ferroalloy.

The mechanism for producing aluminum-silicon-titanium from rich titanium slag by the carbothermic approach (using high-ash coal as a reducing agent) can be described by the dependence of the Gibbs free energy of reduction reactions during smelting. This dependence is shown in Fig. 3.

To obtain a liquid-flowing aluminum-silicon-titanium, initially, an alloy with a high silicon content is smelted. For this purpose, the initial composition of the charge mixture consists only of quartzite and high-ash coal to obtain an intermediate ferro-silicon aluminum alloy. The recovery of silicon follows well-known schemes used for traditional silicon ferroalloys (e.g., ferro-silicon), involving the formation of intermediate products such as carbides (SiC), suboxides (SiO) and the pure silicon (Si). An intermediate compound Al_2_O can be formed during the reduction of aluminum. The charge mixture is loaded with rich titanium slag, reducing the proportion of quartzite in the charge. The process is conducted without slag (slag-free). The reduction of titanium was observed. Thus, it led to formation of titanium silicides through the following reactions (Eqs. (1)–(3)):

[1] $${TiO}_{2}+{SiO}_{2}+4\;C=TiSi+4\;CO$$

[2] $${TiO}_{2}+2\;{SiO}_{2}+6\;C={TiSi}_{2}+6\;CO$$

[3] $$5{TiO}_{2}+3\;{SiO}_{2}+16\;C={Ti}_{5}{Si}_{3}+16\;CO$$

The formation of titanium silicide was further confirmed by X-ray phase analysis of a titanium alloy as a result of the experimental smelting.

The use of rich titanium slag as a titanium-containing material enables an increase in the concentration of titanium in aluminum-silicon-titanium. Simultaneously, the inclusion of quartzite and high-ash coal facilitates a more uniform distribution of the components and enhances their contact surface. It enables a more thorough reduction of titanium, iron, silicon and aluminum from their respective oxides by carbon, while reducing the energy intensity of the process.

## 4 Experimental procedure

Before testing, the electric furnace was heated for 12 h on a coke cushion, which was as a conductor of electric current. After the heating, the electric furnace was completely cleared of residues of the coke cushion. An electrical mode of the heating was performed at a secondary voltage of 24.6 V and a current strength on the high side of 150–200 A.

To melt the charge mixture and form a reaction crucible, a high temperature (up to 3000 K) is achieved due to the arc discharge provided by the graphite electrode.

The main indicators of the periodic melts confirm the possibility to produce the standard titanium rich slag with TiO_2_ content of 75–85%. Smelting was performed periodically, i.e., the charge was loaded after the release and set the current load. The charge was smelted and cured. The slag was tapped out through the overflow lip. The metal was run out through the bottom hole every two releases of slag. After cooling and weighing, each release of slag and metal was chemically analyzed.

**Fig. 4:**
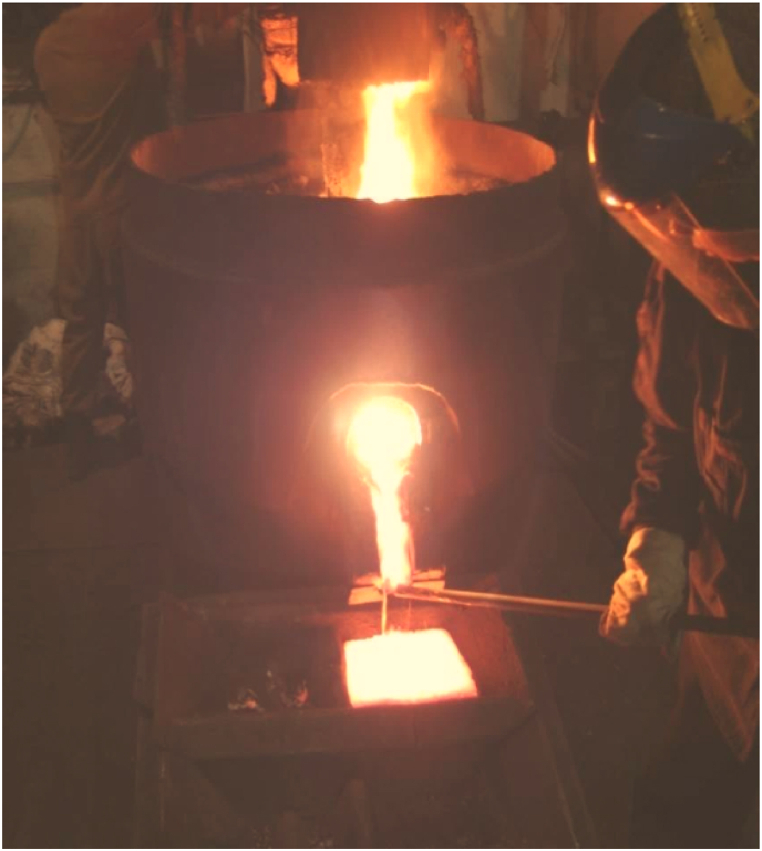
Release of the alloy from furnace.

The titanium-containing ferroalloy was smelted in the same furnace by the continuous method, with loading of the charge in small portions as the top shrinks, and with periodic release of metal every 2 h into the cast-iron molds (Fig. 4).

After 12 h of the heating and coking of the furnace bath, the first charge mixture was loaded. The charge mixture was loaded around the electrode, gradually raising the top. Such loading ensures the uniform filling of the furnace bath, without any sudden jumps in the current load. The notch was opened with an iron rod. The metal of each release was weighed, and then samples were taken for the chemical analysis.

The smelting process was characterized by a stable current regime. In general, the technological process was satisfactory without any violations.

In order to study the features of the produced samples of titanium rich slag and complex alloy, some researches were conducted, including the analysis of the chemical composition, the analysis of the phase composition on X-ray diffractometer Shimadzu XRD-7000, and some metallographic studies. The metallographic studies of the alloy structure were performed on a scanning electron microscope with an energy dispersive spectrometer Carl Zeiss Evo 40. The chemical composition of phases of the alloy was detected using an INCA X-Act energy dispersive spectrometer mounted on a scanning microscope.

## 5 Results and discussion

As a result of the tests, the produced titanium rich slag was characterized by the chemical composition presented in Table 5. The chemical composition of the associated metal is demonstrated in Table 6.

**Table 5:** Average chemical composition of titanium rich slag, %.

Al_2_O_3_	SiО_2_	TiО_2_	Cr_2_O_3_	Fe_2_O_3_	P_2_O_5_	MgO	S	CaO	MnO
4.52	1.98	80.19	1.30	9.87	0.07	3.11	0.021	0.26	1.87

**Table 6:** Average chemical composition of the associated alloyed metal (cast iron),%.

Fe	Cr	Ti	Mn	S	P
95	3.50	0.62	0.15	0.102	0.304

The data of chemical analysis confirm the possibility to produce titanium rich slag with the extraction of the unwanted impurities in the metal.

The associated alloyed metal from titanium slag smelting can be applied as a raw material in the steelmaking.

Then, a new complex titanium-containing ferroalloy produced from titanium rich slag was investigated, where the high-ash coal was used as a reducing agent. The average chemical composition of complex titanium-containing ferroalloy is presented in Table 7.

**Table 7:** Average chemical composition of complex titanium-containing ferroalloy.

Ti	Si	Al	C	P	Fe
20–25	35–45	10–15	0.2–0.5	0.06–0.08	the rest

By the results of chemical analysis, the resulting alloy corresponds to FeT30Al6 (ISO 5454–80) in terms of titanium content.

**Fig. 5:**
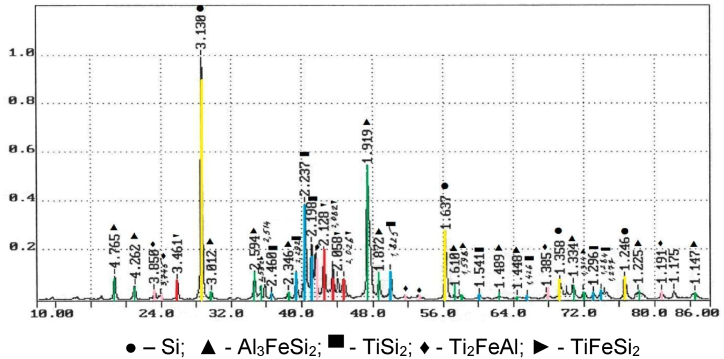
The diffractogram of the produced ferroalloy.

The research of the phase composition on the X-ray diffractometer Shimadzu XRD-7000 is presented as a diffractogram in Fig. 5 and Table 8.

**Table 8:** Results of X-ray phase analysis of the produced complex titanium-containing ferroalloy.

Formula	Mass fraction, %
Si	11
Al_3_FeSi_2_	42
TiSi_2_	25
Ti_2_FeAl	14
TiFeSi_2_	8

The phase analysis detected that in the alloy the active elements (Si, Al, Fe and Ti) are present as complex intermetallides such as Al_3_FeSi_2_, TiSi_2_, Ti_2_FeAl, TiFeSi_2_, and as free silicon.

The metallographic studies of the structure of the ferroalloy sample revealed the composition of the spectrum of the tested pilot complex alloy.

**Fig. 6:**
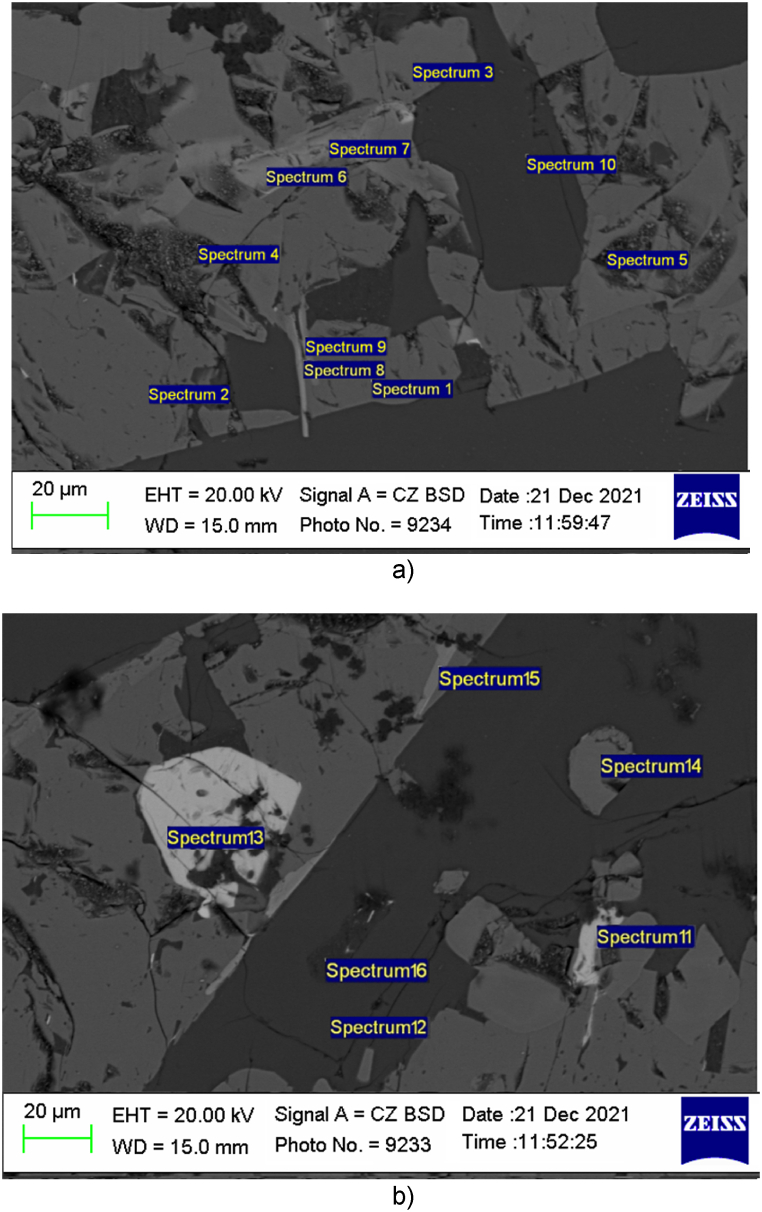
Electronic images of the structure of the produced titanium-containing ferroalloy: a) spectrum 1–10; b) spectrum 11-16.

An image of the microstructure of the titanium-containing ferroalloy obtained on a scanning electron microscope with an energy dispersive spectrometer Carl Zeiss Evo 40 is illustrated in Fig. 6 (a, b).

Table 9 demonstrates the results of the chemical analysis of the] spectra in weight and atomic content.

**Table 9:** Results of chemical analysis of spectra in weight and atomic content.

Spectrum	Content of elements in weight, %				Content of elements in atomic, %			
	Al	Si	Ti	Fe	Al	Si	Ti	Fe
1	67.86	11.32	0.00	20.83	76.42	12.25	0.00	11.33
2	41.90	31.25	0.00	26.85	49.36	35.37	0.00	15.28
3	0.60	52.98	46.42	0.00	0.78	65.54	33.68	0,00
4	2.02	41.07	52.37	4.53	2.75	53.93	40.33	2.99
5	1.41	26.98	32.37	39.23	2.20	40.17	28.26	29.37
6	7.29	52.32	40.39	0.00	9.08	62.59	28.33	0.00
7	12.33	49.66	38.01	0.00	15.13	58.59	26.28	0.00
8	8.36	54.96	36.68	0.00	10.21	64.54	25.25	0.00
9	10.75	48.81	39.14	1.30	13.38	58.39	27.45	0.78
10	0.00	100.00	0.00	0.00	0.00	100.00	0.00	0.00
11	15.10	36.02	0.00	48.88	30.38	69.62	0.00	0.00
12	0.00	100.00	0.00	0.00	0.00	100.00	0.00	0.00
13	29.98	70.02	0.00	0.00	30.82	69.18	0.00	0.00
14	0.00	54.07	45.93	0.00	0.00	66.69	33.31	0.00
15	6.04	52.08	41.89	0.00	7.58	62.81	29.62	0.00
16	99.06	0.94	0.00	0.00	99.08	0.92	0.00	0.00

The microstructure of the alloy detected the presence of two-component compounds of Ti and Si–TiSi and TiSi_2_, which can be found in spectrum of 3, 6, 7, 8, 9, 14 and 15.

The three-component compounds of Si, Ti and Fe are indicated by spectrum of 4 and 5 as white phenocrysts. The phases designated by spectrum of 1, 2, 11 are decoded as intermetallides of Fe, Si and Al. Spectrum of 10 and 12 are presented in dark gray color and occupy a significant area of the sample. Thus, based on the table they are identified as structurally free silicon.

## 6 Conclusions

As a result of the pilot large-scale laboratory researches, two types of the competitive products were produced.

1) Titanium rich slag with the high titanium content was produced from substandard ilmenite concentrate. Its following content of main components (%) was as follows: TiO_2_ - 77.2; Al_2_O_3_ - 4.5; SiO_2_ - 1.97; Cr_2_O_3_ - 1.8 and Fe_2_O_3_ - 9.87.

This technology is used to the ilmenite concentrate tested in this research and to any other concentrate that do not meet the requirements for the content of titanium dioxide and impurities.

2) The complex titanium-containing ferroalloy contents the main components, %: Ti - 20–25; Si - 35–45; Al - 10–15; C – 0.2–0.5; P - no more than 0.08, and ferrum.

This alloy was smelted by the carbothermic approach from titanium rich slag. The high-ash coal (ash content: more than 45%) was applied as a reducing agent. As a result, it was not used for its intended purpose.

The produced alloy corresponds to FeT30Al6 (ISO 5454–80) in terms of titanium content and it can become its full analogue for smelting and alloying of steels.

One ton of the resulting alloy can replace a mechanical mixture for the complex alloying and deoxidizing of steel consisting of 1 ton of FeSi45 ferrosilicon, 150 kg of aluminum shavings, and 1 ton of low-grade FeT30Al6 ferrotitanium. Also, the obtained alloy can become a new complex titanium-containing reducing agent in the smelting of the high-grade ferrotitanium, where titanium rich slag will be applied as a main raw material source.

Thus, the obtained data demonstrate the possibility to use ilmenite concentrates as the main charge materials for smelting of titanium rich slag, even if they do not meet the requirements for the chemical composition. Also the analog of the low-percentage ferrotitanium from titanium rich slag and high-ash coals can be produced. After analyzing the results of this research, it can be concluded that the development of a comprehensive and resource-saving smelting technology, serving as analogues to standard ferroalloy grades was started.

## Funding

The research was conducted within the framework of the study which financially supported by the Scientific Committee of the Ministry of Education and Science of Republic of Kazakhstan (grant No. AP09058310).

## Author contribution statement

N. Vorobkalo: Conceived and designed the experiments; Analyzed and interpreted the data; Wrote the paper. A. Baisanov: Conceived and designed the experiments; Analyzed and interpreted the data. Ye. Makhambetov: Conceived and designed the experiments; Performed the experiments; Analyzed and interpreted the data. Ye. Mynzhasar: Conceived and designed the experiments; Performed the experiments. N. Nurgali: Conceived and designed the experiments; Contributed reagents, materials, analysis tools or data; Analyzed and interpreted the data.

## Data availability statement

The authors are unable or have chosen not to specify which data has been used.

## Declaration of competing interest

The authors declare that they have no known competing financial interests or personal relationships that could have appeared to influence the work reported in this paper.
